# Efficient strain modulation of 2D materials via polymer encapsulation

**DOI:** 10.1038/s41467-020-15023-3

**Published:** 2020-03-02

**Authors:** Zhiwei Li, Yawei Lv, Liwang Ren, Jia Li, Lingan Kong, Yujia Zeng, Quanyang Tao, Ruixia Wu, Huifang Ma, Bei Zhao, Di Wang, Weiqi Dang, Keqiu Chen, Lei Liao, Xidong Duan, Xiangfeng Duan, Yuan Liu

**Affiliations:** 1 Key Laboratory for Micro-Nano Optoelectronic Devices of Ministry of Education, School of Physics and Electronics, Hunan University, Changsha, 410082 China; 2 State Key Laboratory for Chemo/Biosensing and Chemometrics, College of Chemistry and Chemical Engineering, Hunan University, Changsha, 410082 China; 3 Department of Chemistry and Biochemistry, University of California, Los Angeles, CA 90095 USA

**Keywords:** Sensors and biosensors, Electronic properties and materials, Polymers

## Abstract

Strain engineering is a promising method to manipulate the electronic and optical properties of two-dimensional (2D) materials. However, with weak van der Waals interaction, severe slippage between 2D material and substrate could dominate the bending or stretching processes, leading to inefficiency strain transfer. To overcome this limitation, we report a simple strain engineering method by encapsulating the monolayer 2D material in the flexible PVA substrate through spin-coating approach. The strong interaction force between spin-coated PVA and 2D material ensures the mechanical strain can be effectively transferred with negligible slippage or decoupling. By applying uniaxial strain to monolayer MoS_2_, we observe a higher bandgap modulation up to ~300 meV and a highest modulation rate of ~136 meV/%, which is approximate two times improvement compared to previous results achieved. Moreover, this simple strategy could be well extended to other 2D materials such as WS_2_ or WSe_2_, leading to enhanced bandgap modulation.

## Abstract (web summary)

Strain engineering is a promising method to manipulate properties of two-dimensional (2D) materials but slippage between material and substrate makes strain transfer inefficient. Here the authors overcome slipping effects by encapsulating a 2D material in a polymer substrate.

## Introduction

The two-dimensional (2D) materials have attracted considerable attention in recent years, owing to their unique electrical, optical, and mechanical properties at the single atomic thickness^1–8^. From mechanical point of view, 2D materials could withstand deformations over 10% before rupture, which is over one order of magnitude higher than that of typical bulk semiconductors with a break value typically <1% (refs. ^7,9^). The naturally high flexibility has stimulated considerable efforts in further controlling and modulating the electrical and optical properties of 2D semiconductors through strain engineering. For example, by applying tension strains, the bandgap of typical TMD (transition metal dichalcogenide) can be reduced, offering additional degree of freedom to improve the performance of TMD based devices such as strain-induced strong light emission in bilayer WSe_2_ (ref. ^10^), and the greatly enhanced carrier mobilities in strained MoS_2_ transistors^11,12^.

In modern microelectronics, the strain engineering of silicon channel is achieved by doping the source and drain regions with lattice-mismatched atoms such as germanium and carbon^13^. The larger germanium atom provides compression strain to silicon channel with much-enhanced hole carrier mobility, and similarly, smaller carbon atom creates tension strain and is used to increase the electron carrier mobility of silicon. Applying existing state-of-the-art strain approaches to 2D materials is not straightforward because there is little physical space for impurity dopants in such atomically thin semiconductors^14^. Alternatively, in 2D research community, the strain engineering is typically achieved by mechanically exfoliating 2D materials on the surface of flexible polymer substrate that can be mechanically bended or stretched^10,15–19^. However, with weak van der Waals (vdW) force in between^20^, the strain applied on the polymer may not be effectively transferred to the lattice of 2D materials where the decoupling (between polymer and 2D materials) and interlayer slippage are unavoidable^18^, leading to insufficient bandgap modulation. For example, by applying uniaxial strain to monolayer MoS_2_ on PMMA (polymethyl methacrylate) substrate, small bandgap change (Δ*E*_*g*_) of 38 meV and low bandgap modulation rate (*S*_Δ*Eg*_, defined as the slope between Δ*E*_*g*_ and applied strain) ~70 meV/% are observed^21^.

To enhance the Δ*E*_*g*_ and *S*_Δ*Eg*_, considerable efforts have been devoted to increase the strain transfer efficiency from polymer substrate to 2D materials. The early attempt used Ti metal as a clamp to fix the MoS_2_ on the surface of flexible PET (polyethylene terephthalate) substrate to avoid the slippage^22^. However, within this structure, the majority strain may be largely accumulated in the metal–MoS_2_ junction, resulting in nonhomogeneous strain distribution and small bandgap modulation Δ*E*_*g*_ of 110 meV (with *S*_Δ*Eg*_ ~ 45 meV/%) before MoS_2_ rapture. Alternatively, improved strain transfer efficiency can be achieved by using polymers with higher Young’s modulus *E*_Young_, which have been theoretically predicted and experimentally verified^23,24^. For example, by changing the flexible substrate from polydimethylsiloxane (PDMS) (*E*_Young_ ~ 430 kPa) to polyvinyl alcohol (PVA) (*E*_Young_ ~ 650 MPa), the strain transfer efficiency could be improved over 6 fold^18^. However, although increasing the substrate *E*_Young_ could mitigate the Young’s modulus mismatch between 2D materials and substrate, their vdW interaction is still too weak to prevent slippage and interfacial decoupling during straining process, especially at relatively large strain level. Hence using uniaxial strain, the highest bandgap modulation reported for monolayer MoS_2_, WSe_2_, and WS_2_ are limited to 140 meV, 90 meV, 50 meV, respectively^18,25^. The non-optimized bandgap modulation achieved poses a key challenge for investigating fundamental physics of 2D materials through strain engineering, as well as for the practical application of high-performance strain engineered 2D devices and flexible electronics.

Here, we report a simple strain engineering approach to efficiently modulate the bandgap of 2D materials, by encapsulating them in the flexible PVA through spin-coating method. The strong interaction force between the spin-coated PVA and 2D materials, together with the high Young’s modulus of PVA used here (*E*_Young_ ~ 10 GPa)^26^, ensure the mechanical strain applied on the PVA substrate can be effectively transferred to the lattice of 2D materials during the mechanical bending process. By applying uniaxial strain to monolayer MoS_2_, we observed a higher bandgap change of ~300 meV and a highest modulation rate of ~136 meV/%, approximate two times improvement compared to previous best results (Δ*E*_*g*_ ~ 140 meV and *S*_Δ*Eg*_ ~ 70 meV/%, respectively) using uniaxial strain and is approaching the energy required for direct-to-indirect bandgap transition in MoS_2_ (refs. ^18,21^). Furthermore, tape peeling test, thermal expansion measurement, multiple cycles straining-relaxing, and load-unload experiments are conducted to verify the efficient bandgap modulation is the result of strong interaction between MoS_2_ and PVA substrate with negligible slippage in between. Finally, we demonstrate this simple PVA spin-coating approach could be well extended to other TMD such as WS_2_ or WSe_2_, and much higher Δ*E*_*g*_ and *S*_Δ*Eg*_ are observed compared to previous reports. Our study not only breaks the limit of TMD bandgap modulation value using strain engineering, but also provides a general approach for efficient strain engineering of other layered 2D materials and conventional 3D thin films.

## Results

### Device fabrication and straining processes

**Fig. 1:**
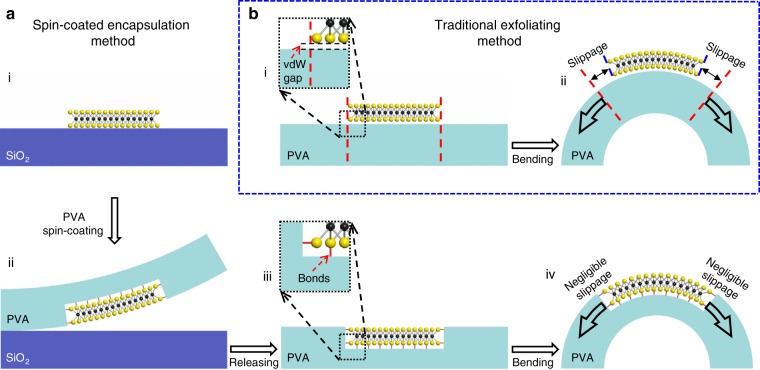
Schematical illustration of fabrication processes of spin-coated encapsulation method and traditional exfoliating method. **a** Schematic fabrication processes of PVA-encapsulation method with four steps: (i) MoS_2_ exfoliation on SiO_2_ substrate, (ii) PVA spin-coating and fully encapsulation of MoS_2_ with strong interaction force in between, (iii) PVA/MoS_2_ released from SiO_2_ substrate, (iv) bended under strain test equipment with negligible slippage. **b** Schematic fabrication processes of traditional exfoliating method with two steps: (i) MoS_2_ direct exfoliation on top of the pre-fabricated PVA substrate, (ii) bended under strain test equipment. Due to the weak vdW force between PVA and MoS_2_, large slippage could happen under tension strain, as highlighted by the distance between the red dash line (ideal position without slippage) and the blue dash line (actual 2D material edge with slippage).

Figure 1 shows the schematic fabrication processes and device structure. To fabricate the device, monolayer MoS_2_ is first mechanically exfoliated on the surface of silicon substrate (p^++^) with 300 nm SiO_2_ on top (Fig. 1a, i). The monolayer thickness of MoS_2_ used here can be confirmed by optical micrograph, Raman spectrum and photoluminescence (PL) spectrum, as shown in Supplementary Fig. 1. Next, PVA layer (with a thickness of ~13 μm) is spin-coated on top of substrate, and fully encapsulates the MoS_2_ underneath, as shown in Fig. 1a, ii. The spin-coated PVA used here could provide strong bonding forces towards underneath MoS_2_ (highlighted by the red links in Fig. 1a), which is essential for efficient strain transfer and will be discussed in detail. After fully encapsulating the MoS_2_ underneath, the PVA and the MoS_2_ are physically released from the SiO_2_ substrate and fixed on two-point apparatus that could continuously apply strain through bending process (Fig. 1a, iii) under a Raman system. When the device is bended, uniaxial tension strain applied on the PVA will be efficiently transferred onto MoS_2_ with negligible slippage owning to their strong interaction, as schematically illustrated in Fig. 1a, iv. The details of apparatus setup and the measurement of strain value are shown in the “Method” section and Supplementary Fig. 2. For comparison, the fabrication processes of control device using conventional direct exfoliation method is also schematically illustrated. As shown in Fig. 1b, i, the monolayer MoS_2_ is mechanically exfoliated on top of pre-fabricated PVA substrate (with same substrate preparation condition as in Fig. 1a), demonstrating low vdW bonding forces towards the PVA substrate. When the strain is applied through mechanically bending, large slippage could happen, as highlighted by the distance between the red dash line (ideal position without slippage) and the blue dash line (actual 2D material edge) in Fig. 1b, ii. The device structures fabricated through both spin-coated encapsulation method and conventional exfoliation method can be further confirmed using atomic force microscopy, as shown in Supplementary Fig. 3.

### Bandgap modulation of monolayer MoS_2_ under uniaxial strain

**Fig. 2:**
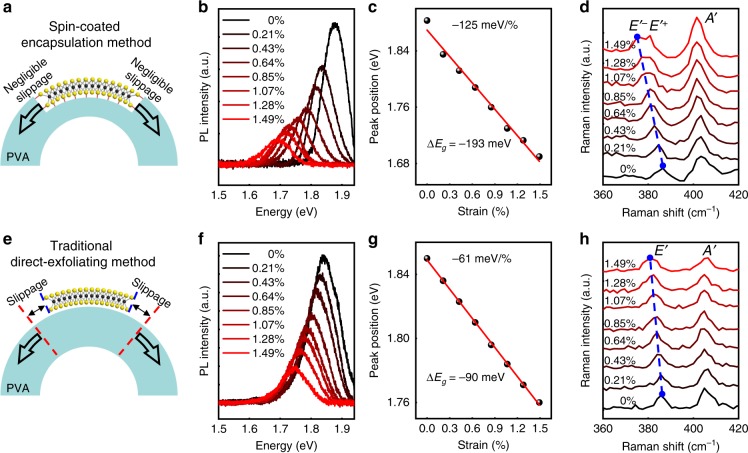
Bandgap modulation of monolayer MoS_2_ using uniaxial tension strain. **a**–**d** PL and Raman spectrums under different mechanical strain using spin-coated PVA encapsulation method. **b** PL spectrum under different tension strain. **c** With applying tension strain up to 1.49%, large bandgap modulation Δ*E*_*g*_ of ~193 meV is observed with a highest modulation efficiency of 125 meV/% using linear fitting (red line). **d** Raman spectrum under different tension strain, and the *E*′^*-*^ peak shows a red shift of 11.1 cm^−1^ with an average slope of about 7.4 cm^−1^/% strain when strain value reaches 1.49%. **e**–**h** PL and Raman spectrums under different mechanical strain using the traditional direct exfoliating method. **f** PL spectrum under different tension strain. **g** With tension strain up to 1.49%, bandgap modulation Δ*E*_*g*_ of ~90 meV is observed with a modulation efficiency of 61 meV/% using linear fitting (red line). The much smaller Δ*E*_*g*_ and slope suggest the inefficient strain transfer from substrate to MoS_2_ using conventional exfoliation approach. **h** Raman spectrum under different tension strain, and the *E*′ peak shows a red shift of 4.8 cm^−1^ with an average slope of about 3.2 cm^−1^/% strain when strain value reaches 1.49%.

We investigate the evolution of the band structure of monolayer MoS_2_ under uniaxial strain through PL and Raman measurement. As shown in Fig. 2a–c, the spectra of the unstrained PVA-encapsulated MoS_2_ shows a prominent A peak at 1.883 eV, suggesting its direct bandgap. Applying tension strain significantly decreases its bandgap (Fig. 2b), and with the strain level of 1.49%, the PL peak value red shifts from 1.883 to 1.690 eV with a Δ*E*_*g*_ of 193 meV, much higher than that previously achieved in monolayer MoS_2_ (Δ*E*_*g*_ ~ 140 meV). The relationship between peak position and the strain value is summarized in Fig. 2c, and the linear fitted strain modulation rate S_Δ*Eg*_ reaches 125 meV/%, which is consistent with 136 meV/% of our density functional theory (DFT) simulation and the previous simulation results^27,28^, as detailed in Supplementary Fig. 4. We note that with further increasing the applying strain value above 1.7%, the strong interaction between MoS_2_ and the substrate may eventually break, leading to the strain relaxation and the device failure, as schematically illustrated and experimentally demonstrated in Supplementary Fig. 5.

Besides the peak position, the peak intensity also reduces with increasing strain, owning to the direct-to-indirect band transition with applying tension strain^22^. Moreover, in order to probe the limit of bandgap modulation and strain tuning capability, we also measure the PL spectrum of the device under compression strain, where highest Δ*E*_*g*_ ~ 300 meV and S_Δ*Eg*_ ~ 136 meV/% are demonstrated, as shown in Supplementary Fig. 6. Besides PL measurement, Raman spectrum is also measured to investigate the lattice change of MoS_2_ device under strain, as shown in Fig. 2d. With the strain value increasing, the *A*′ peak (out-of-plane vibration) keeps relatively constant, and the *E*′ peak (in-plane vibration) redshift towards lower wavenumber, which is expected and consisted with the previous reports^22^. Importantly, as the lattice symmetry is gradually broken, we observe a pronounced *E*′ peak split behavior (into *E*′^*-*^ and *E*′^*+*^, as shown in Fig. 2d), further indicating the applied strain is efficiently transferred to MoS_2_ lattice. When the strain reaches 1.49%, the *E*′^*-*^ peak shows a maximum red shift of 11.1 cm^−1^ with an average slope of about 7.4 cm^−1^/% strain, which is also much higher than previous reports (4.5 cm^−1^/% or 2.1 cm^−1^/%)^22,29^.

To highlight the efficiency of our PVA-encapsulation method, the same bending and spectrums measurements are also applied to our control sample with conventional strain engineering approach, by exfoliating monolayer MoS_2_ on top of pre-fabricated PVA substrate (Figs. 1b and 2e). As shown the in Fig. 2f, g, the exfoliated MoS_2_ demonstrate expected bandgap reduction with applying tension strain. However, due to the weak vdW force between MoS_2_ and the substrate, the strain can not be fully transferred to the lattice of MoS_2_. Hence, small Δ*E*_*g*_ ~ 90 meV, S_Δ*Eg*_ ~ 61 meV/% and Raman shift slope ~3.2 cm^−1^/% strain are observed under the same strain level (1.49%), both of which are less than half of the PVA-encapsulated samples and are consistent with previous reports^18,22^, further suggesting the decoupling between sample and substrate, as well as the inefficient strain transfer using conventional approach. We note the slippage or decoupling effect may not only exist in uniaxial experiment (through substrate bending), but could also happen for biaxial straining of 2D materials using AFM tip based or micro-chamber based blister or balloon experiments owning to the weak vdW interaction between 2D material and the anchor substrate during the chamber pressure change, as have been reported in previous literatures^30–32^.

Furthermore, the demonstrated effective strain modulation is a robust behavior within different locations of given sample, as well as between different samples. To demonstrate this, we have applied PL mapping to monolayer MoS_2_ samples fabricated through both our spin-coated encapsulation method, as well as conventional exfoliation approach. As shown in Supplementary Fig. 7, for conventional exfoliation sample with weak MoS_2_–PVA interaction, the PL peaks show visible peak fluctuation (~30 meV), consistent with previous literature^16^ and the highest bandgap modulation is ~60 meV under strain of 1.28%. In contrast, the device fabricated through our PVA encapsulation approach shows more uniform PL peak (with negligible variation) and the bandgap modulation value is ~120 meV, which is around two times improvement compared to conventional exfoliation method. Furthermore, we have also measured multiple devices to show the consistency of our data, and to produce the confidence interval for the measured parameters. As shown in Supplementary Fig. 8, for our spin-coating encapsulation method, the maximum modulation value is in the range of 190 meV to 240 meV using tension strain, and the modulation efficiency is in the range of 125 meV/% to 131 meV/%. In contrast, for devices using conventional exfoliation approach, the maximum modulation value is in the range of 90 meV to 110 meV, and the modulation efficiency is in the range of 44 meV/% to 63 meV/%.

### Mechanisms of the efficient strain transfer

The efficient bandgap modulation achieved above could be attributed to three advantages of our PVA encapsulation method. First, the most functional groups of PVA are hydroxyl groups^33^, and therefore could provide a stronger adhesion force towards the MoS_2_ than typical PDMS or PET substrate used. In fact, PVA itself has been widely used as adhesive glue in the textile industry owing to its excellent adhesion properties^34,35^. Secondly, the spin-coating method ensures the conformal contact between PVA and MoS_2_ and the possible chemical bonds at the defects points and edge sidewall of MoS_2_ (as highlighted in Fig. 1a, iii), which is much stronger compared to the conventional approaches with weak vdW bonding forces^33,36^. It has been reported that the sulfur vacancy^37^ is the primary defect of MoS_2_ and the defect density could reach as high as 6.5 × 10^13^/cm^2^, which would provide enough dangling-bonds to form strong interaction with spin-coated PVA. To confirm this, we have further applied tape peeling test, as shown in Supplementary Fig. 9. For convention MoS_2_ exfoliated on top of pre-fabricated PVA substrate with weak vdW interaction, there is no sample left on PVA anymore after peeling by tapes (Supplementary Fig. 9c, f). In great contrast, MoS_2_ flakes encapsulated in PVA can pass this peeling test and remains on PVA substrate after repeated peeling using different tapes (Supplementary Fig. 9h-k), suggesting stronger interaction force between MoS_2_ and PVA (compared to that of MoS_2_ and tapes). Thirdly, the PVA used here have a larger *E*_Young_ ~ 10 GPa, which is much higher than typical used PDMS with *E*_Young_ of 430 KPa. The high *E*_Young_ here is essential for efficient strain transfer to MoS_2_, as have been theoretically analyzed using finite element simulation (Supplementary Fig. 10) and experimentally demonstrated in our samples with different substrate *E*_Young_ (Supplementary Fig. 11). Together with strong interaction force (to hold PVA and MoS_2_) and high substrate *E*_Young_, the simple spin-coated PVA encapsulation method used here could provide near unity strain transfer efficiency, as can also be quantitively and directly measured using optical microscope through thermal expansion experiment (Supplementary Fig. 12).

### Multi-cycles load and unload test

**Fig. 3:**
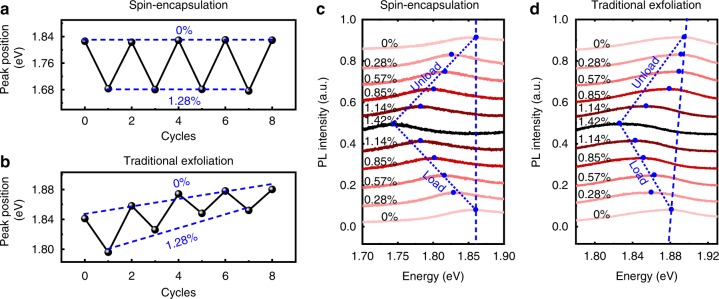
Multiple cycles straining-relaxing and load-unload bending test. **a**, **b** The multiple cycles straining and relaxing at fixed tension strain of 1.28%, for devices fabricated by our PVA encapsulation method (**a**) and the conventional exfoliating approach (**b**). Within spin-coated PVA approach (**a**), the PL spectrums demonstrate repeatable peak position under multiple cycles test, suggesting the negligible slippage and efficient strain transfer. In contrast, within conventional exfoliating approach (**b**), the PL spectrums demonstrate a much smaller Δ*E*_*g*_ and a gradual blue shift with increasing bending cycles, indicating the poor strain transfer and a significant slippage behavior. **c**, **d** The load-unload bending test under variable tension strain level, for our PVA encapsulation method (**c**) and the conventional exfoliating approach (**d**). Within spin-coated PVA approach (**c**), the PL spectrums demonstrate repeatable peak position change, further confirming the negligible slippage and efficient strain transfer. In contrast, within conventional exfoliating approach (**d**), the PL spectrums demonstrate much smaller modulation efficiency and blue shift of peak position during unloading process, indicating that the poor strain transfer and a significant slippage behavior.

To further confirm the high strain transfer efficiency and negligible material slippage, multiple cycles straining-relaxing test is conducted. Figure 3a, b show the extracted PL peak position as a function of the repeated cycles of straining and relaxing, and strain value is fixed at 1.28%. For our PVA-encapsulated sample, the PL emission peak always comes back to the same value between each cycle, indicating that the MoS_2_ flake does not slip during the measurement and the applied strain is successfully transferred to the lattice of MoS_2_, as highlighted by the blue dash line (Fig. 3a). In great contrast, the control sample of exfoliated MoS_2_ (on pre-fabricated PVA substrate) shows much smaller Δ*E*_*g*_ with applying same level of strain, as shown in Fig. 3b. More importantly, with increasing the straining-relaxing cycles, the PL peak of control sample gradually blueshift and can not back to the same value between each cycle (highlighted by the blue dash line in Fig. 3b), indicating the material slippage and the substrate decoupling dominate the whole bending process. Such small non-repeatable PL spectrum (within weakly bonded system) have also been observed in previous studies^24,38^, which may be attributed to the finite in-plane compression during the unloading process, as shown in Supplementary Fig. 13.

Furthermore, the minimized material slippage can also be confirmed by load-unload test, where continuous strain is applied and withdrawn from the sample. As shown in Fig. 3c, d, for our spin-coated encapsulation sample, the unloading process shows identical PL spectrums with the loading process (Fig. 3c, blue dash line), where the PL peak can go back to its original value under different strain level. In contrast, for direct exfoliated control sample, very small bandgap changes, and non-repeatable PL spectrums are observed, further confirming the MoS_2_ slippage and inefficient strain transfer during bending process.

### Bandgap modulation for a variety of other monolayer 2D materials

**Fig. 4:**
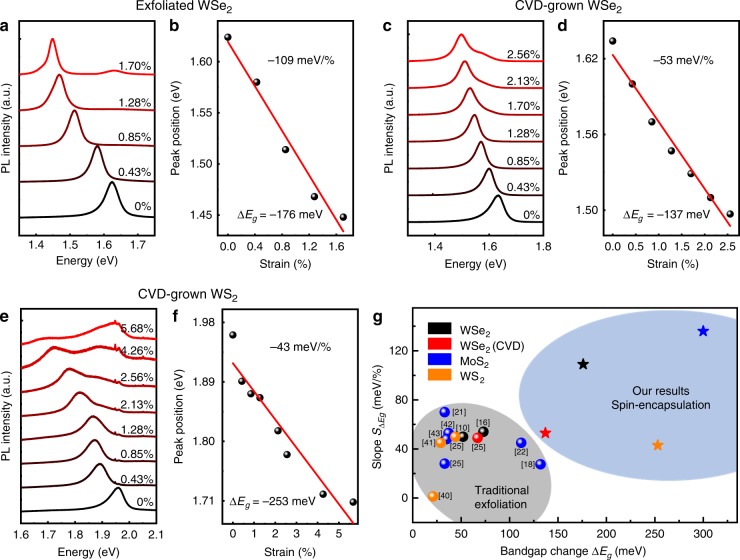
Photoluminescence measurement under different tension strain of other TMD materials using PVA encapsulation approach. **a**, **b** PL spectrum of mechanically exfoliated monolayer WSe_2_ under difference strain (**a**). The bandgap change Δ*E*_*g*_ is 176 meV with a linear fitted slope of 109 meV/% (**b**). **c**, **d** PL spectrum of the monolayer CVD-grown WSe_2_ under difference strain (**c**). The bandgap change Δ*E*_*g*_ is 137 meV with a linear fitted slope of 53 meV/% (**d**). **e**, **f** PL spectrum of monolayer CVD-grown WS_2_ under difference strain (**e**). The bandgap change Δ*E*_*g*_ is 253 meV with a linear fitted slope of 43 meV/% (**f**). **g** The bandgap strain modulation of different materials and different approaches. Our spin-encapsulation method (highlighted by blue area) provides higher Δ*E*_*g*_ (*x*-axis) and *S*_Δ*Eg*_ (*y*-axis), compared to that of the traditional exfoliation method (highlighted by gray area).

The simple spin-coated PVA encapsulation approach is not only limited to MoS_2_, but could be well-extended to other 2D semiconductors to achieve higher bandgap modulation than typical exfoliation approach. To demonstrate this, we have applied this simple method to mechanically exfoliated monolayer WSe_2_, CVD (chemical vapor deposition) grown monolayer WSe_2_, as well as CVD grown monolayer WS_2_. As shown in Fig. 4a–f, all of these materials demonstrate distinct bandgap reduction with applying uniaxial tension strain, consistent with MoS_2_ and previous reports. The peak position can be further extracted and plotted as a function of the strain applied. As shown in Fig. 4b, d, f, highest Δ*E*_*g*_ of 176 meV (*S*_Δ*Eg*_ = 109 meV/%), 137 meV (*S*_Δ*Eg*_ = 53 meV/%), 253 meV (*S*_Δ*Eg*_ = 43 meV/%) are observed for exfoliated WSe_2_, CVD WSe_2_, and CVD WS_2_, respectively. The difference between exfoliated WSe_2_ and CVD grown WSe_2_ could be attributed to the inferior quality of CVD grown samples, with possible dopants, defects, or pre-strains. Nevertheless, the observed Δ*E*_*g*_ values are much higher than previous reports^10,16,18,21,22,25,39–44^, indicating our method is a general approach for effective strain transfer with negligible slippage.

At last, we compare the performance of our PVA-encapsulation approach with previous strain engineering methods in terms of Δ*E*_*g*_ and *S*_Δ*Eg*_. As shown in Fig. 4g, our spin-coated PVA encapsulation approach demonstrates higher Δ*E*_*g*_ and *S*_Δ*Eg*_, as highlighted by blue area, and is much higher than various strain engineering methods in previous reports (highlighted by the gray area). This suggests that our simple strain engineering approach may be further extended to other emerging 2D materials or thin film semiconductors, with another degree of freedom to further manipulate their band structures, as well as electronic transport properties (e.g., high mobilities in Hall bar structure, or lower contact resistance in transistor). Furthermore, we note the efficient straining of vdW heterostructures would be an interesting topic for future investigation. However, our spin-coating encapsulation method only provide strong interaction between the PVA substrate and one layer of 2D material, while the heterostructures are still bonded through weak vdW interaction and the interlayer slippage may dominate the overall device straining behavior.

## Discussion

In summary, we report a simple and efficient strain engineering approach to modulate the bandgap of monolayer 2D materials by using PVA encapsulation through a spin-coating method. The strong adhesion force (between the spin-coated PVA and 2D materials) and high *E*_Young_ of PVA ensure the mechanical strain can be effectively transferred to the lattice of 2D materials. By applying uniaxial strain to monolayer MoS_2_, we observed a higher bandgap modulation Δ*E*_*g*_ ~ 300 meV and *S*_Δ*Eg*_ ~ 136 meV/%, which is approximately two times enhancement compared to previous best results, and is consistent with our DFT calculations. Furthermore, we confirmed the negligible slippage (between TMD and spin-coated PVA) in our system through detailed tape peeling test, multiple cycles straining-relaxing, thermal expansion measurement, and load-unload bending experiments. Our simple method offers a general strain engineering approach beyond the limit conventional direct-exfoliating method, providing another degree-of-freedom to discover and to investigate the fundamental physics in 2D layered materials as well as conventional 3D thin film materials. It may also provide exciting implications for electronic, optoelectronics, nanoelectromechanical systems, and other devices that can benefit from the flexible, transparent nature of atomic monolayers.

## Methods

### PVA substrate preparation

For PVA encapsulation method, monolayer MoS_2_ is first mechanically exfoliated on the surface of the silicon substrate (p^++^) with 300 nm SiO_2_ on top (substrate size: 30 × 10 mm^2^). Next, 10 wt% PVA (Alfa Aesar, 98–99% hydrolyzed, molecular weight 130,000 g/mol) solution is spin-coated on the surface of SiO_2_ (with MoS_2_) at a speed of 1000 rpm for 40 s, and baked at 70 °C (1 min) to remove water solvent. Subsequently, a PET (Shanghai Feixia Rubber and Plastic Hardware Trading Co., Ltd, 125 μm thick) film is glued onto the upper surface of the PVA film using adhesive glue (Shenzhen Kaibingtuan Plastic Industry Co., Ltd, 5562 instant adhesive) to increase the thickness for easily handling of flexible substrate. Next, the entire flexible substrate (PVA/glue/PET) with the encapsulated MoS_2_ can be slowly peeled off from SiO_2_ substrate using tweezer, as shown in the Fig. 1a, ii. The PVA film prepared here is measured to be ~13 μm and entire flexible substrate is measured to be ~185 μm (PVA/glue/PET), as shown in the schematic and cross-section optical image in Supplementary Fig. 2a, b.

### Calculation of tension strain values

In general, the strain applied on flexible substrate can be expressed through the simple equation *ε* = *τ*/*R*, where 2*τ* and *R* are the substrate thickness and curvature radius, respectively. The thickness 2*τ* of the entire flexible substrate is accurately measured to be ~185 μm through the cross-section optical image, as shown in Supplementary Fig. 2a, b. Therefore, the measurement of bending curvature is the key factor for the substrate strain calculation. To accurately measure the actual curvature radius *R*, we have imaged the curvature radius through both the cross-sectional optical image and photograph, and directly measured the accurate curvature radius *R* under the device, as shown red dash line in Supplementary Fig. 2c–e (fitted using commercial software Digimizer).

### First-principles calculations

The first-principles calculation is carried out by the open-source QUANTUM ESPRESSO plane-wave density functional theory (DFT) package^45,46^. The Perdew-Burke-Ernzerhof (PBE) exchange-correlation functional with semi-empirical DFT-D3 vdW correction method is adopted^47^ and the plane-wave cut-off energy is 1115 eV. To avoid mirror interactions, a vacuum space of 15 Å is added between adjacent cells in the thickness direction. The Brillouin zone *k*-point sampling is 10 × 10 × 1 for electronic ground-state computations. The crystal structures are fully relaxed until the force on each atom and total energy variations are smaller than 2.6 × 10^−2^ eV/Å and 1.4 × 10^−3^ eV (ref. ^48^). The strains are achieved by varying the relaxed cell parameters and the atom positions are optimized again after the variations.

### 3D finite element (FE) simulation

The discretized FE models contain about 12,099 units with very fine meshes in the contact region. The final mesh density was determined through a series of convergence studies. Appropriate boundary conditions were used along two edges of the PVA to simulate the loading strain. The interface between MoS_2_ and PVA were modeled using perfect bonding. The calculations were performed using commercial FE package COMSOL Multiphysics (version 5.2). Young’s modulus and Poisson’s ratio are 10 GPa and 0.3 for PVA substrate, 170 GPa and 0.27 for MoS_2_ nanosheet, respectively.

### Raman and PL measurement

The entire mechanical device and flexible substrate are placed on a confocal microscope (Renishaw invia-reflex) to measure Raman and photoluminescence spectrums. For PL spectrum measurement, 532 nm laser with 1800 lines mm^−1^ grating is used, where laser power is 500 μW (MoS_2_), 50 μW (WSe_2_) and 500 μW (WS_2_), respectively. For Raman spectrum measurement, 488 nm laser is used with 250 μW power and a grating levels of 2400 lines mm^−1^.

## Supplementary information

Supplementary Information

## Data Availability

The data that support the findings of this study are available from the corresponding author upon reasonable request.
