# Supplementary material for: Efficient strain modulation of 2D materials via polymer encapsulation
Source: Nat Commun. 2020 Mar 2;11:1151. doi: 10.1038/s41467-020-15023-3 (PMC7052151; doi:10.1038/s41467-020-15023-3)
Supplement: Supplementary file 1 — Supplementary Information [file 41467_2020_15023_MOESM1_ESM.pdf]

Supplementary Information

Efficient strain modulation of 2D materials via polymer encapsulation

Li et al.

Contents:

Supplementary Figures 1-13
Supplementary References

Supplementary Figures

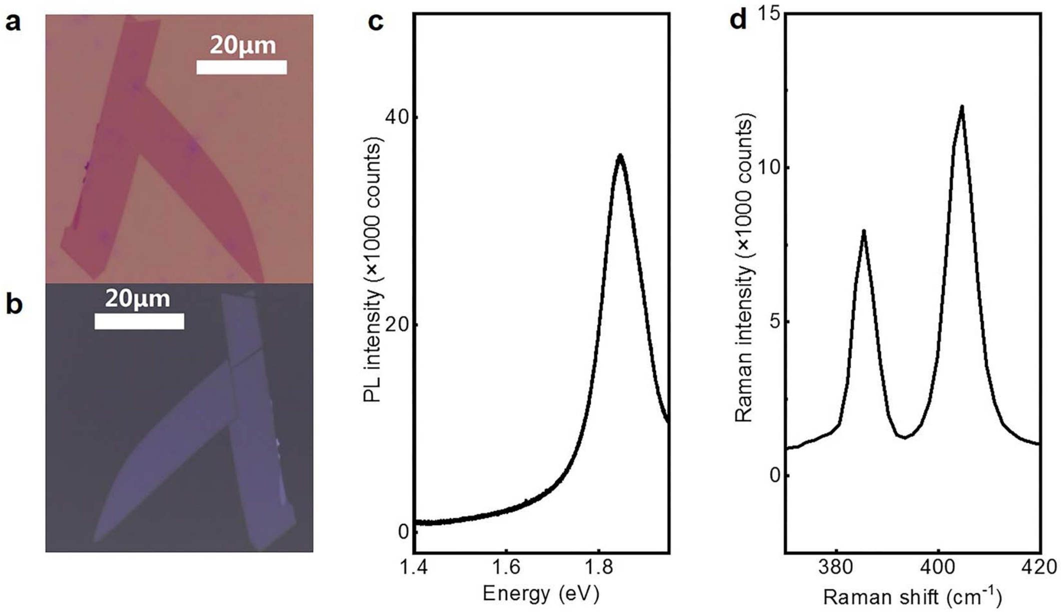

Supplementary Figure 1. The optical image, PL spectrum and Raman spectrum of monolayer MoS_2. a, The optical image of exfoliated monolayer MoS_2 on SiO_2 substrate. **b**, The optical image of monolayer MoS_2 after encapsulated by spin-coated PVA. **c**,**d**, The PL (**c**) and Raman (**d**) spectrum of exfoliated monolayer MoS_2 encapsulated inside PVA.

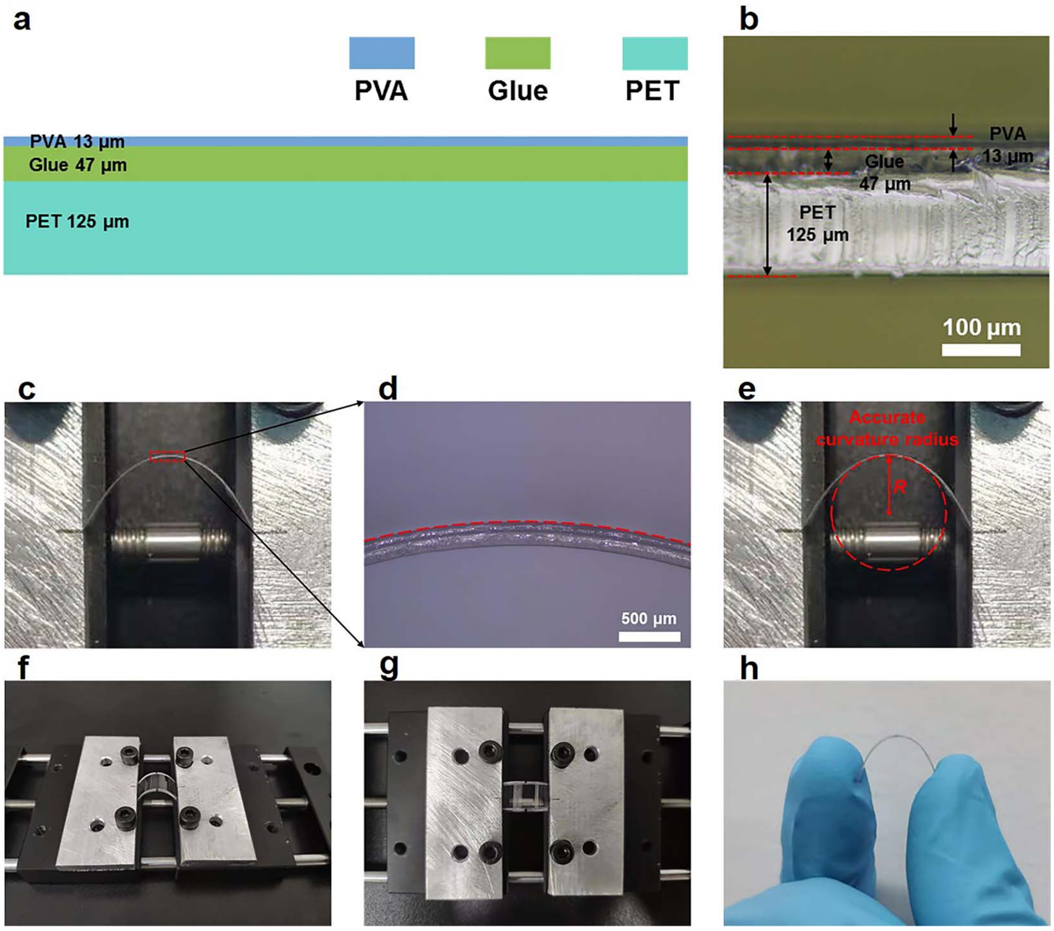

Supplementary Figure 2. **Measurement of substrate thickness and curvature radius, and photography of bend apparatus**. **a**, Schematic diagram of the entire substrate (PVA/glue/PET) with thickness labeled. **b**, The cross-section optical image of the flexible substrate, where the thicknesses of PVA, glue and PET are measured to be 13 μm, 47 μm and 125 μm, respectively. **c**, Cross-section image of the bent substrate. **d**, Zoomed-in optical image of the center region in the bent substrate, and the red dotted arc indicates the software (image analysis software Digimizer) fitting path. **e**, The accurately measured curvature radius *R* (red dotted arc) in the cross-section view. **f**-**h**, Photographs of the bending apparatus.

AFM measurement

In order to illustrate the actual structures and confirm their consistency with our schematic illustrations in Fig. 1. We have applied atomic force microscopy (AFM)

measurement of the MoS_2-PVA structure fabricated by both our PVA encapsulation and traditional exfoliation methods, as is shown in Supplementary Figure 3 below. For the PVA-encapsulated structure, there is no height different (<0.2 nm) between, but only phase different between the PVA region and the MoS_2 region (Supplementary Figure 3c,d), suggesting the MoS_2 flake is fully encapsulated by the spin-coated PVA and is consistent with schematics in Fig. 1. In contrast, for device fabricated through typical exfoliation method, obvious height and phase difference are observed between PVA and MoS_2 region (Supplementary Figure 3g,h), which is consistent with direct exfoliation of 2D materials on various pre-fabricated substrate (*e.g.*, SiO_2). We note applying AFM measurement on soft substrate (PVA in our case) tends to show low resolution due to the smaller Young's modulus, which will mask the true height information of the monolayer MoS_2 (<1 nm), hence, we use multi-layer MoS_2 (5-12 nm) samples for the AFM measurement, which won't impact the demonstration of structure difference between PVA encapsulated device and conventional exfoliated device.

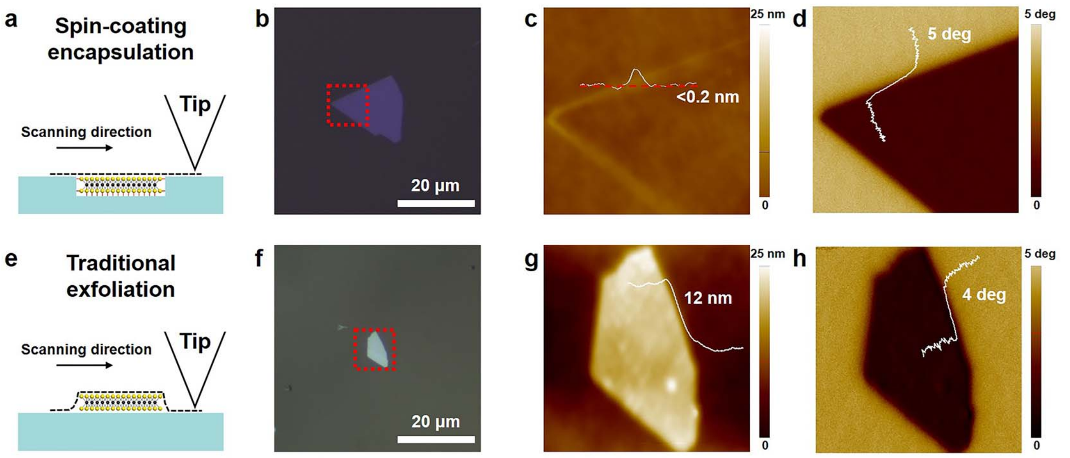

Supplementary Figure 3. Atomic force microscopy measurement of the MoS_2-PVA structure fabricated by both methods. **a**, AFM measurement schematic of the MoS_2-PVA fabricated by our spin-coating encapsulation method. **b**, Optical image of MoS_2 flake (thickness ~5 nm) encapsulated by PVA. **c**,**d,** AFM topography (**c**) and phase image (**d**) of red rectangular area in **b**. No height difference (<0.2 nm) and only phase difference is

observed between MoS_2 and PVA region, suggesting the MoS_2 flake is fully encapsulated and is consistence with the schematics in **a**. **e**, AFM measurement schematic of the MoS_2-PVA fabricated by typical direct exfoliation method. **f**, Optical image of MoS_2 flake (thickness 12 nm) exfoliated on PVA. **g**,**h**, AFM topography (**g**) and phase image (**h**) of red rectangular area in **f**. Both height and phase difference are observed between MoS_2 and PVA, consistent with the schematics in **e**.

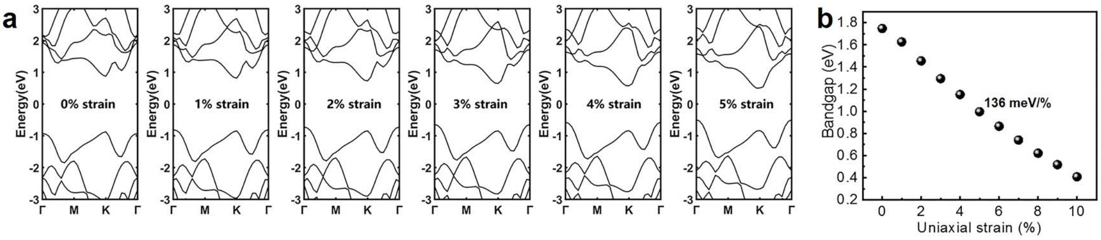

Supplementary Figure 4. DFT simulation result of monolayer MoS_2 with uniaxial tension strain (alone Zigzag direction). **a**, Band structures of monolayer MoS_2 for different strains. **b**,The bandgap reduces with increasing strain, with a slope of 136 meV/%.

Device failure mode and its mechanism

For our encapsulated devices, the strong interaction force and high modulation rate are achieved through the intimate contact and possible chemical bonds between 2D materials and the spin-coated PVA substrate. With increasing the applying strain value above a threshold value, these chemical bonds may eventually break, leading to the relaxation of strain for the 2D materials (with vdW contact towards the substrate) and the device failure, as schematically illustrated in the Supplementary Figure 5a-c. This process can be further confirmed using PL measurement. As shown in Supplementary Figure 5d,e, the device shows linearly PL peak shift from 1.88 eV to 1.69 eV with strain from 0% to 1.49%. With further increasing the strain above 1.7%, the PL peak changes back to 1.81 eV, suggesting the strain relax and device failure.

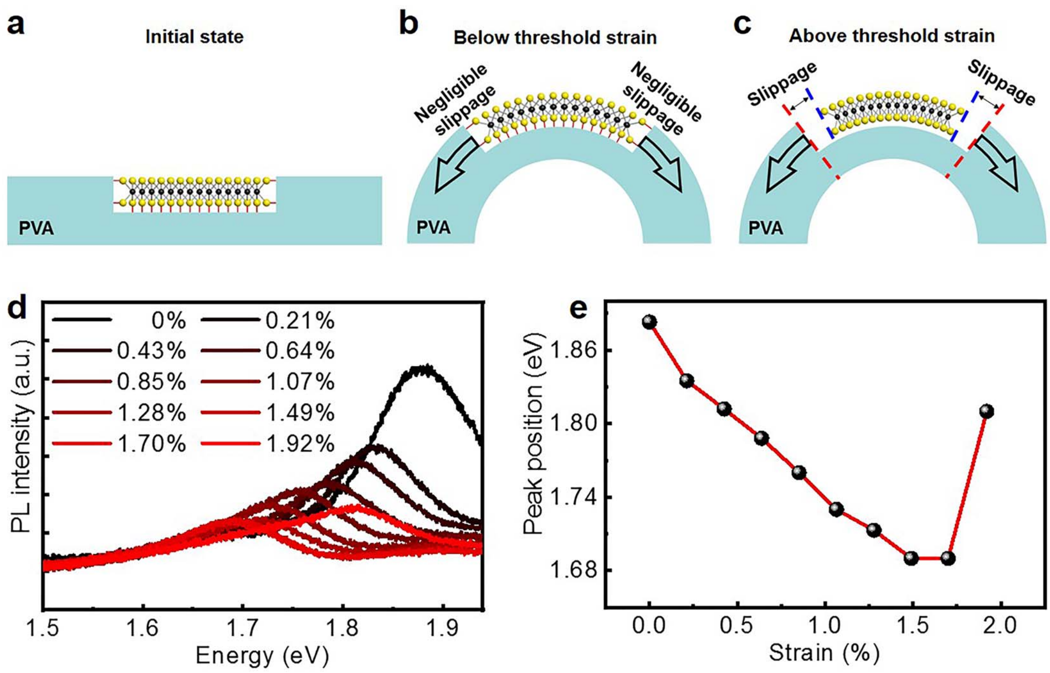

Supplementary Figure 5. Bandgap modulation of monolayer MoS_2 using uniaxial tension strain. **a**,**b**, Schematic diagram of substrate in initial state (**a**), and under strain below threshold value (**b**). **c**, Schematic diagram of substrate bending above the threshold strain, with possible chemical bonds broken, leading to the relaxation of strain for the MoS_2 and the slippage between MoS_2 and the PVA substrate. **d**, PL spectrum under different tension strain. **e**, With applying tension strain up to 1.49%, PL peak linearly shift from 1.88 eV to 1.69 eV. With further increasing the strain above 1.7%, the PL peak changes back to 1.81 eV, suggesting the strain relax and device failure.

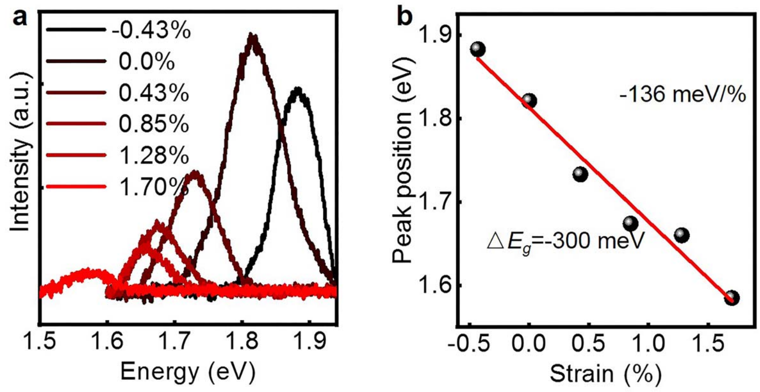

Supplementary Figure 6. Photoluminescence spectrum of mechanically exfoliated

monolayer MoS_2 under compression and tensile strain using PVA encapsulation method. a, PL spectrum under different compression and tension strain. **b**, With strain from -0.43% to 1.7%, largest bandgap modulation ΔE_g of ~300 meV is observed with a modulation rate of ~136 meV/% using linear fitting (red line).

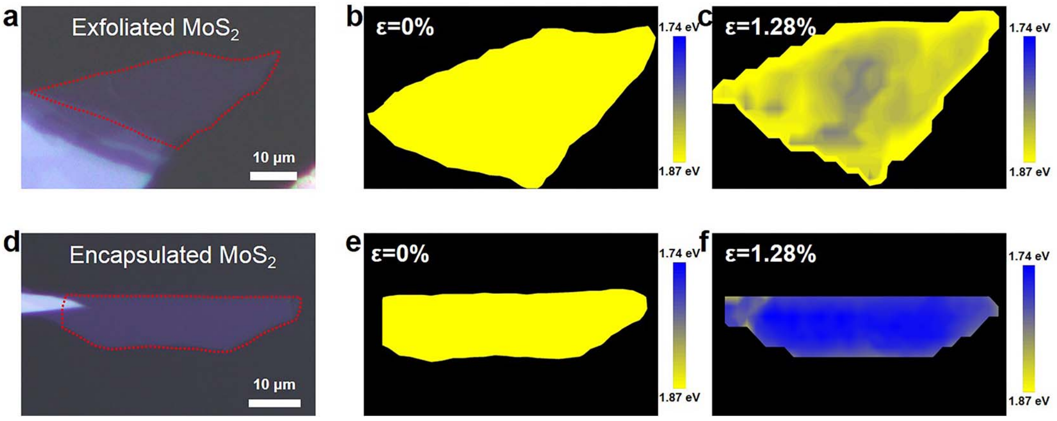

Supplementary Figure 7. PL peak mapping of monolayer MoS_2 using uniaxial tension strain. a-**c**, PL peak mapping of monolayer MoS_2 on top of PVA using conventional exfoliation approach. **a**, Optical image of monolayer MoS_2 (highlighted by red dotted line). **b**, PL peak mapping of MoS_2 without strain, the peak positions are majorly located at 1.84 eV. **c**, PL peak mapping of MoS_2 with applying 1.28% strain, the peak position distribution is in the range of 1.78~1.81 eV. **d**-**f**, PL peak mapping of monolayer MoS_2 encapsulated in PVA using our spin-coating approach. **d**, Optical image of monolayer MoS_2 (red dotted line). **e**, PL peak mapping of MoS_2 without strain, the peak positions are majorly located at 1.86 eV. **f**, PL peak mapping of MoS_2 with applying 1.28% strain, the peak positions are shifted to 1.74 eV, with better uniformity compared to that using exfoliation method.

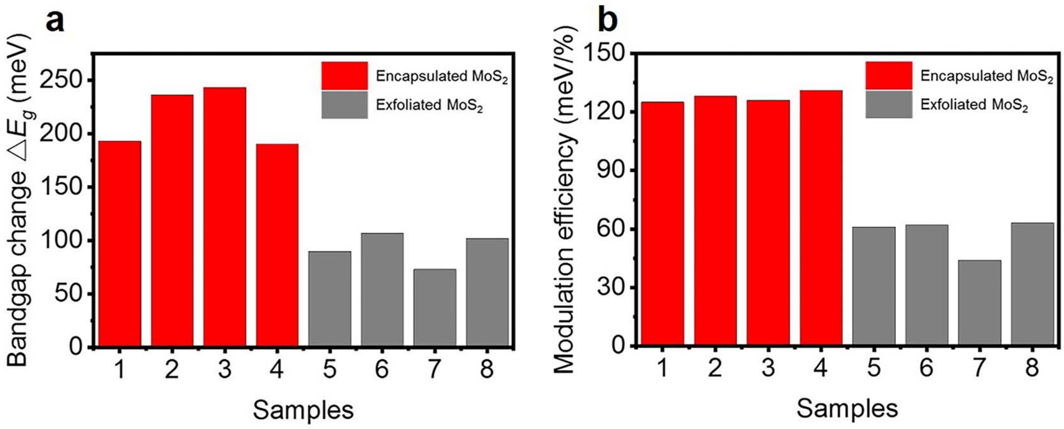

Supplementary Figure 8. The confidence interval for bandgap change and modulation efficiency. **a**, Bandgap change (ΔE_g) is distributed in 190-240 meV for four encapsulated MoS_2 devices, and is distributed in 80-110 meV for devices with conventional exfoliation method. **b**, Modulation rate ($S_{\Delta Eg}$) is distributed in 125-131 meV/% for four encapsulated MoS_2 devices, and is distributed in 44-63 meV/% for devices with conventional exfoliation method.

Tape peeling test

Tape peeling test is a simple mechanical test method of the interaction force between a sample and a given substrate, by using typical tape. Here we use two commonly used tapes Nitto SPV 224 and Scotch Tape 810, that have distinct adhesion strength of 0.55 N/cm and 8 N/cm (calibrated to steel), respectively. For convention MoS_2 exfoliated on top of PVA substrate with weak vdW interaction (Supplementary Figure 9b,e), there is no sample left on PVA anymore after peeling by both tapes (Supplementary Figure 9c,f), and these samples can be found on the tape side (Supplementary Figure 9d,g), indicating the smaller interaction force between MoS_2 and PVA (compared to that of MoS_2 and tapes). In great contrast, MoS_2 flakes encapsulated in PVA can pass this peeling test and remains on PVA substrate after peeling by both tapes, as shown in Supplementary Figure 9h-k, suggesting stronger interaction force between MoS_2 and

PVA.

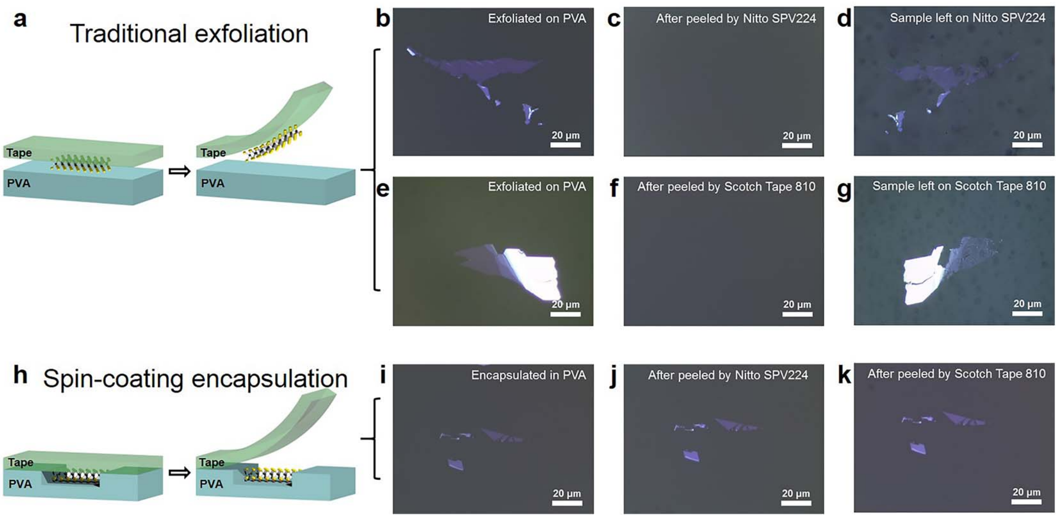

Supplementary Figure 9. Tape peeling test of the MoS_2-PVA structure fabricated by both methods. a-g, Schematics and optical images of tape peeling test in conventional exfoliated MoS_2-PVA interface, using two typical tapes Nitto SPV 224 and Scotch Tape 810. There is no sample left on PVA anymore after peeling by tapes (**c**,**f**), and these samples can be found on the tape side (**d**,**g**), indicating the weaker interaction force between MoS_2 and PVA (compared to that of MoS_2 and tapes). **h-k**, MoS_2 flakes encapsulated in PVA can pass this peeling test and remains on PVA substrate after peeling by tapes, suggesting stronger interaction force between MoS_2 and PVA.

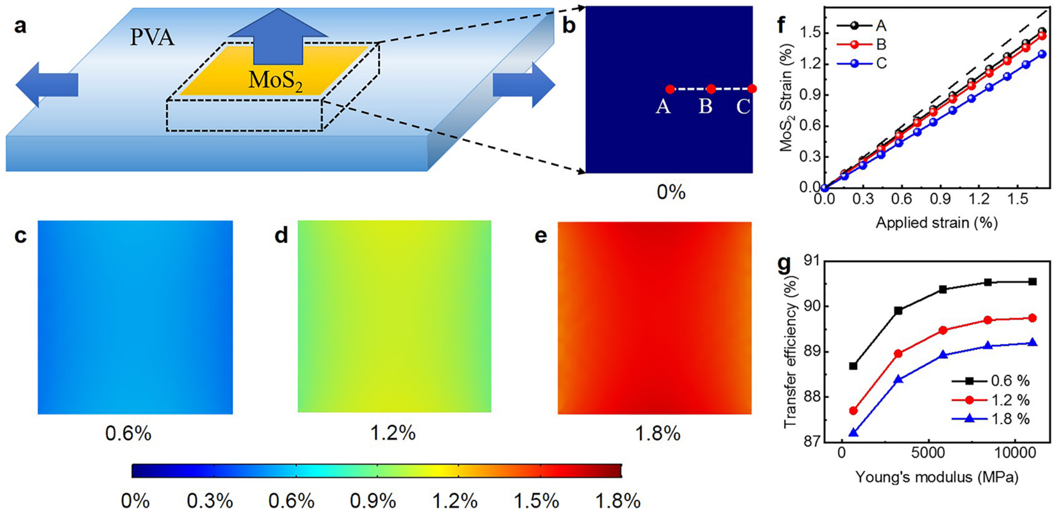

Supplementary Figure 10. FE simulation of tensile-strained MoS_2. a, FE model of the tensile test sample consisting of substrate (PVA) and a monolayer MoS_2 (0.7 nm)

encapsulated in the PVA substrate. **b-e**, Snapshots of FE simulation of MoS_2 tensile tests: applied strain (**b**) 0%, (**c**) 0.6%, (**d**) 1.2%, and (**e**) 1.8%. **f**, Strain in MoS_2 layer as a function of the applied strain on the substrate at points A, B, C. **g**, Strain transfer efficiency in monolayer MoS_2 layer as a function of substrate's Young's modulus for 0.6%, 1.2% and 1.8% tensile strain, where the substrate strain can be effectively transferred onto the monolayer MoS_2.

Effect of substrate Young's modulus on bandgap modulation.

To investigate the impact of substrate Young's modulus, we have measured the MoS_2 devices exfoliated on different substrates using conventional direct-exfoliation method, as shown in Supplementary Figure 11a below. The device exfoliated on PDMS substrate shows much smaller modulation rate (7 meV/%) compared to device exfoliated on pre-fabricated PVA substrate (46 meV/%), suggesting the high E_{Young} is important to improve the strain transfer rate, which is consistent with previous report[1].

Furthermore, we have also investigated the impact of the PVA molecular weight (MW) used for our spin-coating encapsulation method. We have spin-coated the PVA with lower molecular weight (MW) of ~31,000 g/mol (E_{Young} ~2.3 GPa) onto monolayer MoS_2, and measured the strain modulation rate, as shown in Supplementary Figure 11b below. The device exhibits similar strain modulation rate (120 meV/%) compared to our measurement results using high molecular weight of 130,000 g/mol (E_{Young} ~10 GPa), which is consistent with our FE simulation in Supplementary Figure 10g.

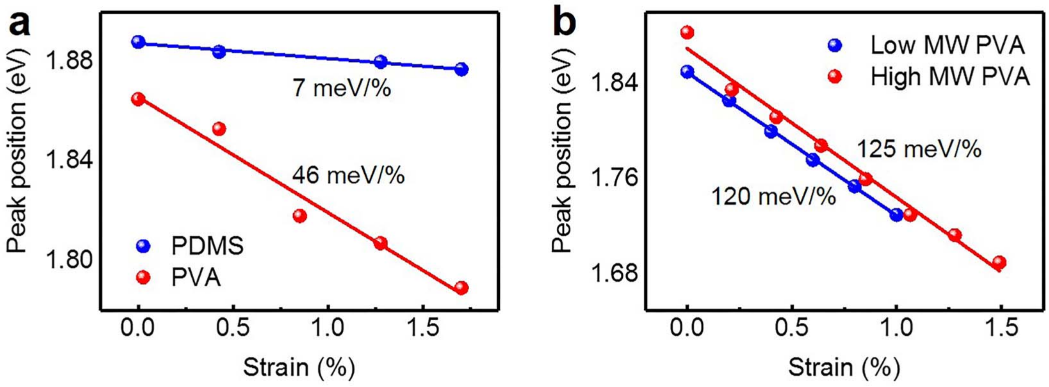

Supplementary Figure 11. Bandgap modulation of monolayer MoS_2 with different substrates and different methods. a, Bandgap modulation using pre-fabricated PVA substrate (high E_{Young} of 10 GPa) and PDMS substrate (low E_{Young} of 430 kPa) through conventional exfoliation method. With applying a tension strain up to 1.7%, bandgap modulation efficiency of ~7 meV/% is observed with device exfoliated on PDMS substrate using linear fitting (blue line), demonstrating a lower modulation efficiency compared with device exfoliated on high E_{Young} PVA substrate (red line) and suggesting the high substrate E_{Young} is important for strain transfer. **b**, Bandgap modulation using PVA substrate with low MW (31,000 g/mol) through our spin-coating encapsulation method. With applying a tension strain up to 1.0%, bandgap modulation efficiency of ~120 meV/% is observed using linear fitting (blue line), demonstrating a similar modulation efficiency compared with device fabricated through spin-coating high MW (130,000 g/mol) PVA (red line).

Thermal expansion experiment

During the thermal expansion experiment, both the PVA encapsulated samples (using CVD grown WSe_2) and the conventional direct exfoliated samples (2D material on pre-fabricated PVA) are fabricated using previously described methods, as shown in the schematics in Supplementary Figure 12a,b. The use of CVD grown material here is essential to directly measure the strain induced size change, owning to its relatively large flake size (~100 μm). Next, the PVA substrate is glued on a PDMS substrate (with

a thickness of ~1.5 mm) and heated on a ceramic heater under microscope, and the thermal induced expansion of PVA surface is measured by the distance change between two gold markers (Supplementary Figure 12a-f) on top of PVA. As shown in Supplementary Figure 12g,h below, the PVA expanded ~1.8% to 2.3% with temperature increasing from 30 ˚C to 120 ˚C, suggesting a tension strain generated. Furthermore, the expansion of 2D material can also be directly measured through optical images. For control sample with conventional exfoliation method, the graphene flake expanded from 117.9 μm to 118.2 μm with a strain of 0.25% (Supplementary Figure 12c,e), suggesting an inefficiency strain transfer efficiency inside the weakly coupled system, as shown in Supplementary Figure 12g. In contrast, within our spin-coating encapsulation method, with PVA substrate strain of 1.82%, the WSe_2 flake expanded from 83.5 μm to 85.0 μm (Supplementary Figure 12d,f) with a similar strain of ~1.80%, suggesting near unity transfer efficiency using our encapsulation approach, as shown in Supplementary Figure 12h.

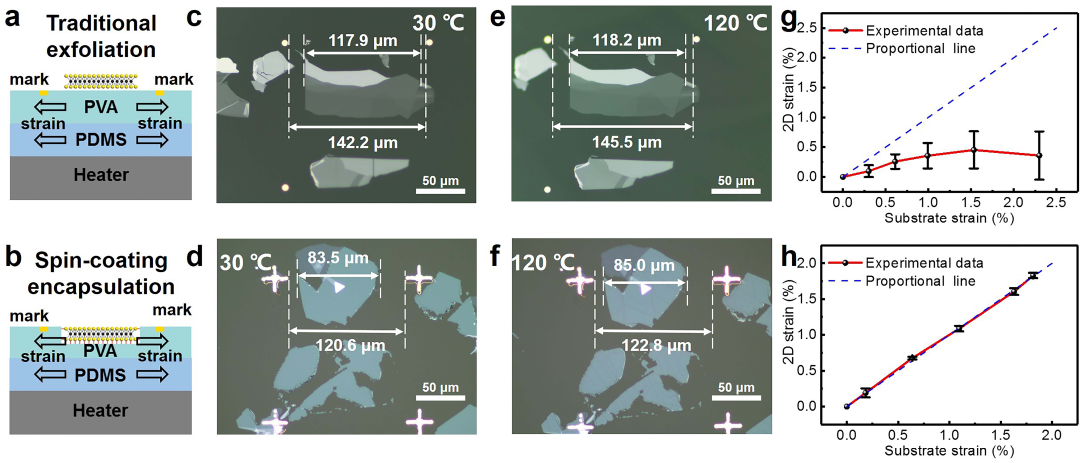

Supplementary Figure 12. Thermal expansion experiment of the 2D-PVA structure fabricated by both methods. a,c,e,g, Thermal expansion of 2D material (graphene) using conventional exfoliation method (with weak vdW interaction). With increasing temperature to 120 ˚C, tensional strain ~2.32% is generated inside PVA due to thermal expansion, as calibrated by gold marker (golden solid dots). While the graphene flake

expanded from 117.9 μm to 118.2 μm with a strain of 0.25%, suggesting an inefficiency strain transfer efficiency. **b**,**d**,**f**,**h**, Thermal expansion of 2D material (CVD WSe_2) using our spin-coating method. With increasing temperature to 120 ˚C, the WSe_2 flake expanded from 83.5 μm to 85.0 μm with a strain of 1.80%, similar as strain inside the PVA substrate (calibrated by golden cross marker) with a value of 1.82%, suggesting near unity transfer efficiency using our encapsulation approach. Data points and error bars represent the mean and standard deviation respectively for each sample.

Non-repeatable PL spectrums for traditional exfoliation method

In traditional exfoliation method with vdW interaction forces between 2D materials and substrate, the 2D materials would slip during the tensile deformation. Although the vdW force is not strong enough to prevent the slippage during the loading process, it is enough to provide little in-plane compression during releasing or unloading steps[2,3], as schematically illustrated in Supplementary Figure 13a-c. Therefore, the PL peak position will normally blueshift a bit (due to the compression) compared to its initial state (Supplementary Figure 13a) and yields a non-reproducible strain cycle.

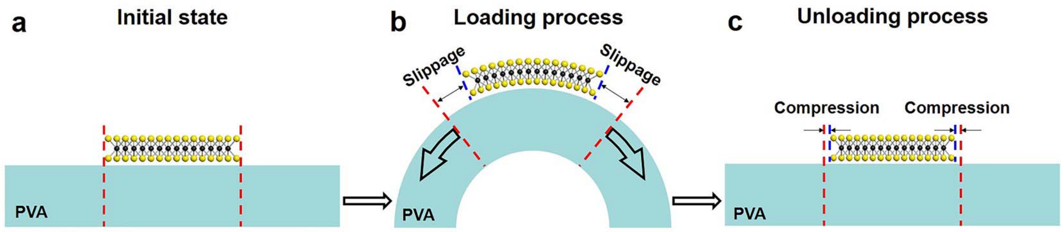

Supplementary Figure 13. Schematically illustration of non-repeatable straining process for traditional exfoliation method. a, Initial state schematic diagram of mechanically exfoliated MoS_2 on PVA substrate. **b**. Schematic diagram of loading/stretching process, the distance between the red (ideal edge position without slippage) and blue dashed lines (actually edge position) indicates the slippage between 2D material and the substrate. **c**, Schematic diagram of unloading/releasing process, the distance between the red (ideal edge position without slippage) and blue dashed lines

(actually edge position) indicates the resulting in-plane compression.

Supplementary References

1. Liu, Z. *et al.* Strain and structure heterogeneity in MoS_2 atomic layers grown by chemical vapour deposition. *Nat. Commun.* **5**, 5246 (2014).
2. Gong, L. *et al.* Interfacial stress transfer in a graphene monolayer nanocomposite. *Adv. Mater*. **22**, 2694-2697 (2010).
3. Jiang, T., Huang, R. & Zhu, Y. Interfacial sliding and buckling of monolayer graphene on a stretchable substrate. *Adv. Funct. Mater*. **24**, 396-402 (2014).
